# Geroscience and pathology: a new frontier in understanding age-related diseases

**DOI:** 10.3389/pore.2024.1611623

**Published:** 2024-02-23

**Authors:** Monika Fekete, David Major, Agnes Feher, Vince Fazekas-Pongor, Andrea Lehoczki

**Affiliations:** ^1^ Department of Public Health, Semmelweis University, Budapest, Hungary; ^2^ Departments of Hematology and Stem Cell Transplantation, South Pest Central Hospital, National Institute of Hematology and Infectious Diseases, Saint Ladislaus Campus, Budapest, Hungary

**Keywords:** geroscience, senescence, cancer, cardiovascular disease, ageing

## Abstract

Geroscience, a burgeoning discipline at the intersection of aging and disease, aims to unravel the intricate relationship between the aging process and pathogenesis of age-related diseases. This paper explores the pivotal role played by geroscience in reshaping our understanding of pathology, with a particular focus on age-related diseases. These diseases, spanning cardiovascular and cerebrovascular disorders, malignancies, and neurodegenerative conditions, significantly contribute to the morbidity and mortality of older individuals. We delve into the fundamental cellular and molecular mechanisms underpinning aging, including mitochondrial dysfunction and cellular senescence, and elucidate their profound implications for the pathogenesis of various age-related diseases. Emphasis is placed on the importance of assessing key biomarkers of aging and biological age within the realm of pathology. We also scrutinize the interplay between cellular senescence and cancer biology as a central area of focus, underscoring its paramount significance in contemporary pathological research. Moreover, we shed light on the integration of anti-aging interventions that target fundamental aging processes, such as senolytics, mitochondria-targeted treatments, and interventions that influence epigenetic regulation within the domain of pathology research. In conclusion, the integration of geroscience concepts into pathological research heralds a transformative paradigm shift in our understanding of disease pathogenesis and promises breakthroughs in disease prevention and treatment.

## Introduction

Geroscience, an emerging discipline at the intersection of aging and disease, seeks to understand the relationship between the aging process and pathogenesis of age-related diseases [1, 2]. Historically, the concept of cellular pathology laid the groundwork for modern pathology [3]. Today, geroscience promises to revolutionize this field by illuminating how aging mechanisms contribute to the pathogenesis of diseases associated with aging [4]. Geroscience has evolved significantly, fueled by discoveries that suggest that aging is governed by evolutionarily conserved cellular and molecular mechanisms and is a modifiable process. This perspective offers a novel lens through which the field of pathology can be viewed. Cellular aging is now viewed as a complex, regulated and potentially reversible process, governed by a genetic program, transcending the mere accumulation of macromolecular damage over time [4]. It is intricately intertwined with cellular dysfunction, playing a pivotal role in the onset of age-associated diseases. This perspective highlights that aging is not just a passive decline in cellular integrity but a coordinated series of events that might be amenable to targeted interventions.

Expanding on the core principles of geroscience, it’s important to delve into the fundamental, evolutionarily conserved aging mechanisms. These mechanisms, often referred to as the “pillars” or “hallmarks” of aging, drive the functional and phenotypic changes in aging cells [5]. Understanding these mechanisms is crucial for both geroscience and pathology, as they provide insights into the biological processes that underlie aging and age-related diseases. These mechanisms play a critical role in multitude of age-related diseases, including hematological diseases [6, 7], Alzheimer’s disease [8–12], osteoporosis, sarcopenia, various types of cancer, presbycusis and cardiovascular and cerebrovascular diseases [13–21]. In pathology, this link is crucial for understanding the etiology and progression of diseases that demonstrate an age-related increase in morbidity and mortality.

## Hallmarks of aging and their pathological implications

**FIGURE 1:**
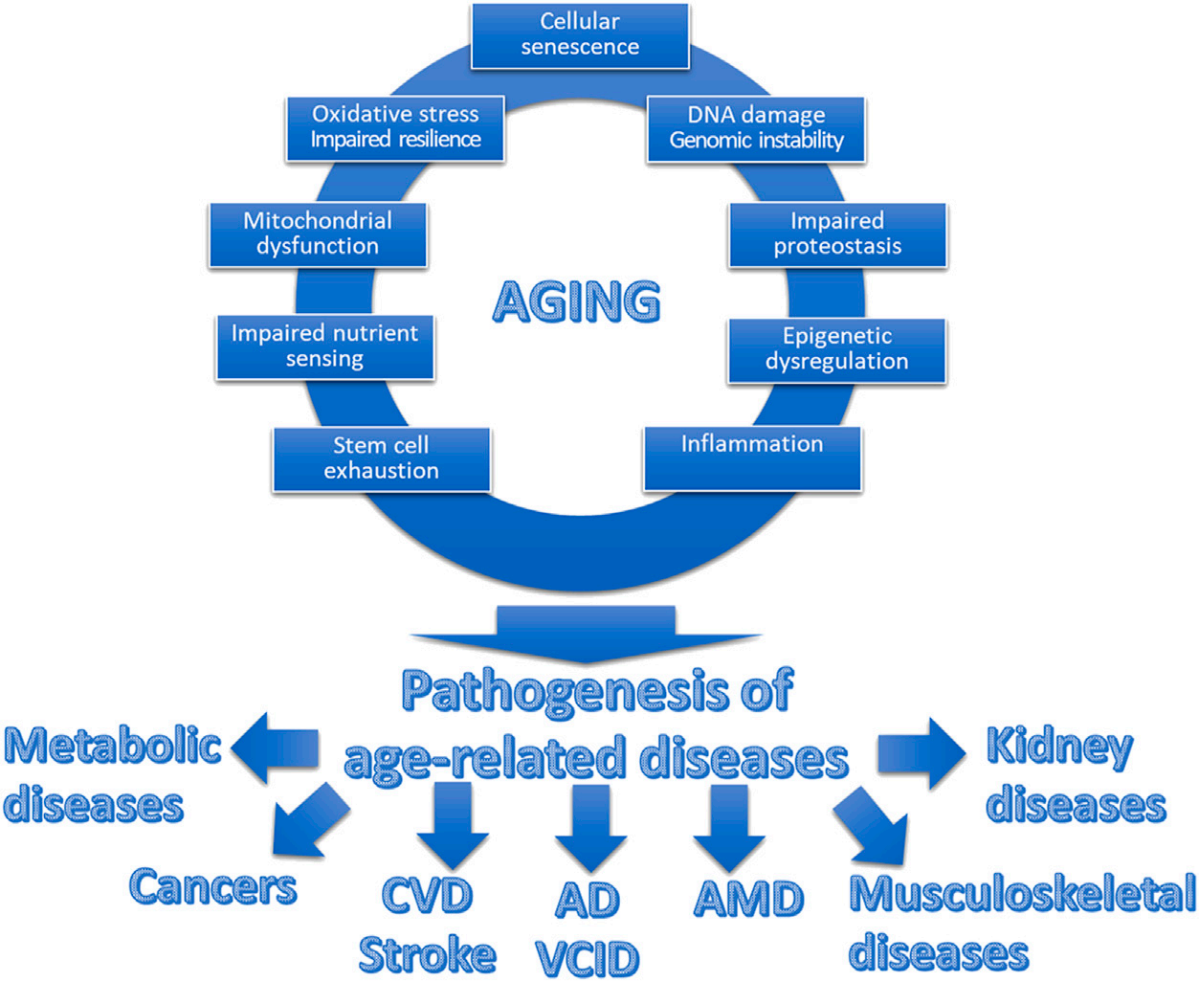
Interplay between hallmarks of aging and age-related diseases. This schematic figure illustrates the interplay between the fundamental cellular and molecular mechanisms of aging, collectively contributing to what we perceive as “aging.” The top section showcases these interconnected hallmarks of aging, encompassing processes such as cellular senescence, mitochondrial dysfunction, genomic instability, and heightened state of inflammation, among others. These synergistic aging processes promote the pathogenesis of a spectrum of diverse age-related diseases. These diseases, including but not limited to cardiovascular diseases (CVD), cancer, neurodegenerative disorders (including Alzheimer’s disease; AD), vascular cognitive impairment and dementia VCID), eye diseases (including age-related macular degeneration, AMD), musculoskeletal diseases and metabolic diseases, share a common origin in the aging process.

Understanding the role of mechanisms/hallmarks of aging is pivotal in pathology, especially when examining age-related diseases [10, 22, 23]. Each of these aging mechanisms is intricately interconnected, collectively contributing to the pathology of a diverse array of age-related diseases (Figure 1). Their combined effects underscore the complexity of aging as a multi-faceted process, deeply influencing the onset and progression of various pathologies. Generally, a significant number of these mechanisms stem from spontaneous, stochastic damage, which in turn activates evolutionarily conserved cellular responses such as cellular senescence and inflammatory pathways. Concurrently, these processes are intertwined with pathways that determine cellular resilience to such damage or stress, including but not limited to the maintenance of proteostasis [24–28], the efficacy of Nrf2-driven antioxidant responses [29–36], and the integrity of DNA repair systems [37].

Recent advancements have revealed that cell-autonomous mechanisms alone do not fully account for the complexities of aging. Instead, aging of various organ systems is also governed by a hierarchical regulatory cascade, where cell-autonomous aging processes are modulated by systemic and circulating factors [28, 38–40]. Furthermore, age-related changes within cells often lead to non-cell autonomous effects, such as the release of paracrine mediators from senescent cells, which in turn influence tissue aging [41–43]. A unifying model suggests that the orchestration of aging processes across various organs involves a balance of circulating pro-geronic and anti-geronic factors. These factors, emanating from the central nervous system, endocrine organs, the immune system, adipose tissue, and even the gastrointestinal tract, play pivotal roles in orchestrating and harmonizing aging processes. An important implication of this concept is that conventional risk factors (including lifestyle [44–50] and environmental risk factors [51, 52]) exacerbate age-related pathologies by intensifying fundamental molecular and cellular aging processes, both cell autonomous and non-cell autonomous.

Since the free radical theory of aging was published in the 1950s, oxidative stress has been increasingly recognized as a key player in the aging process [53, 54]. While reactive oxygen species (ROS) play essential roles in cell signaling, excessive ROS production or inadequate ROS detoxification can lead to oxidative stress, damaging cells and tissues and causing cellular dysfunction [55–60]. In the context of aging, the balance between ROS production and antioxidant defenses becomes skewed. Age-related increases in oxidative stress are thought to contribute to cardiovascular pathologies [61–71], the deterioration of neurological function and development of brain and muscle aging phenotypes, among others. One of the most critical impacts of oxidative stress in aging is on cellular signaling pathways, including triggering of inflammatory mechanisms [30, 67].

The reduced capacity of older cells to counter molecular stress and achieve homeostasis is a critical aging phenomenon [72]. In youthful organisms, a rise in cellular reactive oxygen species prompts adaptive homeostatic responses, chiefly through the activation of the Nrf2 (Nuclear factor erythroid 2-related factor 2)-driven antioxidant defense pathway [30–32, 73]. Nrf2, a transcription factor sensitive to redox changes, coordinates this response, up-regulating the levels of enzymes that neutralize ROS and repair ROS-inflicted macromolecular damage [74]. This adaptive response effectively mitigates oxidative stress in younger cells. However, as organisms age, a significant reduction in Nrf2 functionality occurs, especially noticeable in vascular tissues. This reduction intensifies oxidative stress and increases the susceptibility of aged cells to ROS-related damage, potentially playing a pivotal role in the emergence of age-related pathologies [30, 31]. Notably, the beneficial anti-aging effects associated with caloric restriction are linked to the stimulation of these Nrf2-mediated pathways [29, 75].

Genomic instability, characterized by the accumulation of DNA damage from environmental stressors and metabolic activities, is a key contributor to aging [76]. This instability can result in mutations and chromosomal aberrations, which are central to the development of many age-related diseases, particularly cancer and cardiovascular diseases [18, 77, 78]. Recently, clonal hematopoiesis of indeterminate potential (CHIP) has emerged as a significant area of interest in geroscience [79–88]. CHIP refers to an age-related phenomenon where hematopoietic stem cells acquire somatic mutations that lead to pre-malignant clonal expansion of white blood cells [81–88]. These cells exhibit marked changes in their function, including a pro-inflammatory shift in their phenotype. Importantly, CHIP is not necessarily linked to hematological malignancies but is associated with an increased risk of developing a range of age-related diseases, including atherosclerosis [81–88].

Telomeres, which are protective caps at the ends of chromosomes, gradually shorten with each cell division [18, 89]. Excessive shortening contribute to cellular aging or apoptosis, impairing tissue repair and regeneration [90–93]. Telomere attrition is thought to contribute to many age-related diseases and conditions [18, 94–97].

Age-related changes in epigenetic regulation, such as DNA methylation [94, 98–103], affect gene expression and are implicated in dysregulation of various cellular processes with age and the pathogenesis of age-related diseases, including cancer [104], cardiovascular diseases [105] and Alzheimer’s disease.

With aging, the ability of cells to maintain protein homeostasis diminishes [106], leading to the accumulation of misfolded proteins [107]. Impaired protein homeostasis has been linked to the pathogenesis of a wide range of age-related diseases and conditions [108]. This loss is characteristic of neurodegenerative diseases like Parkinson’s disease and Alzheimer’s disease, where protein aggregates are a hallmark feature [109], disrupting normal organ function.

Aging affects nutrient-sensing pathways [110], impacting metabolism and cellular growth. Alterations in insulin signaling and mTOR pathways are considered evolutionarily conserved mechanisms of aging [111–113]. These changes are evident in age-related diseases like type 2 diabetes and are implicated in the pathogenesis of many age-related diseases, including various types of cancer [114–117] cardiovascular diseases and Alzheimer’s disease.

Autophagy, a multifaceted cellular pathway, stands as a central player in the intricate interplay between aging and disease processes [118–121]. It plays a pivotal role in maintaining cellular homeostasis by degrading and recycling damaged or dysfunctional cellular components, thereby promoting cellular renewal and survival. In the context of aging, autophagy’s efficacy tends to decline, leading to the accumulation of cellular debris and dysfunctional organelles, which are hallmarks of aging [122–125]. The influence of autophagy extends beyond cellular maintenance, as it significantly impacts immune regulation [126, 127] and tumorigenesis [128]. One of the critical regulatory connections involving autophagy is its interaction with the mTOR pathway [129, 130]. mTOR is a central regulator of cellular growth and metabolism, and it inhibits autophagy when nutrients are plentiful. Conversely, during nutrient scarcity or stress conditions, mTOR inhibition allows autophagy to proceed, promoting the removal of damaged cellular components and recycling of resources. This intricate balance between mTOR and autophagy is intimately tied to aging and disease [129, 130], as excessive mTOR activity and compromised autophagy are associated with age-related pathologies, including neurodegenerative diseases and cancer. In the context of immune regulation, autophagy plays a dual role [126, 127]. It contributes to the activation and functioning of immune cells, aiding in the presentation of antigens. Conversely, autophagy can also regulate immune responses, influencing immune tolerance and autoimmunity. Dysregulation of autophagy in immune cells can have profound consequences on an individual’s susceptibility to infections and autoimmune diseases [131]. Regarding tumorigenesis, autophagy’s role is complex [128]. It can promote tumor survival under certain conditions by providing cancer cells with nutrients during nutrient deprivation or stress. In summary, autophagy represents a multifaceted cellular pathway with far-reaching implications in aging. Its intricate relationship with mTOR highlights its significance in aging and disease processes, making it a compelling focus of research in the field of geroscience and cancer biology. Understanding how to modulate autophagy effectively may hold promise in promoting healthy aging and combating age-related diseases.

Sirtuins, a family of NAD+-dependent protein deacetylases, have emerged as pivotal regulators of cellular and organismal aging [132–140]. By modulating the acetylation status of a myriad of protein targets, Sirtuins regulate cellular metabolism, genomic stability, stress resilience and stress response pathways [141–150]. Through their influence on key cellular processes such as DNA repair, mitochondrial function, and inflammation, Sirtuins directly impact the rate of aging and age-related diseases [141–150]. Their intricate involvement in pathways related to calorie restriction and cellular homeostasis has led to substantial interest in their potential as therapeutic targets for extending healthspan and combating age-related diseases. Cytoplasmic (SIRT1, SIRT2, SIRT6) [151, 152], nuclear (SIRT1, SIRT2, SIRT6), nucleolar (SIRT7) [153], and mitochondrial (SIRT3, SIRT4, SIRT5) [154–158] sirtuins represent distinct subtypes within the broader family of sirtuins. SIRT1, the most extensively studied cytoplasmic sirtuin, is known for its specific roles in modulating cellular responses to nutrient availability and energy balance. It plays a crucial role in calorie restriction-related pathways, where it promotes the deacetylation and activation of various transcription factors, such as PGC-1α and FOXO, which are involved in metabolic processes, mitochondrial biogenesis, and antioxidant defense. SIRT2 regulates cytoskeletal dynamics and cell cycle progression, while SIRT6 and SIRT7 are implicated in DNA repair and chromatin maintenance, contributing to genomic stability. SIRT3, for example, promotes the deacetylation and activation of several mitochondrial proteins involved in oxidative phosphorylation and antioxidant defense, thereby enhancing mitochondrial efficiency and reducing oxidative stress. SIRT4 and SIRT5 play roles in regulating mitochondrial metabolism and amino acid metabolism, respectively. Dysregulation of cytoplasmic, nucleolar, or mitochondrial sirtuins can contribute to cellular dysfunction and accelerate the aging process, making them intriguing targets for interventions aimed at promoting healthy aging and mitigating age-related diseases.

Mitochondrial dysfunction is also a hallmark of aging [5, 159–161], impairing cellular energy metabolism and increasing oxidative stress, which profoundly affect cellular health and function. Mitochondrial dysfunction is thought to contribute to a range of age-related diseases [162], including neurodegenerative disorders [163], cardiovascular diseases [67, 72, 75, 164–166] and musculoskeletal diseases [167], including sarcopenia [168].

In aging dividing cells often enter a state of cellular senescence, impacting tissue function [169]. Cellular senescence is a DNA damage-induced cellular stress response, which is associated with profound changes in cellular phenotype [14]. Senescent cells secrete a range of inflammatory factors [170] and enzymes disrupting the extracellular matrix, contributing to tissue aging and dysfunction [171]. The accumulation of senescent cells is thought to be a driver of aging [111, 172–174] and have been linked to the pathogenesis of a number of age-related diseases [175], including musculoskeletal diseases [176, 177], neurodenegenenerative diseases [8, 10, 178] and VCI [179, 180], retina diseases [181] and cardiovascular diseases. The increased presence of senescent cells, often a result of DNA damage induced by lifestyle, environmental exposures [182], and certain interventions like anti-cancer therapies [183, 184], is believed to lead to accelerated aging, exacerbating the progression of age-related conditions and physiological decline. The intersection of cellular senescence biology and cancer biology represents a highly dynamic and increasingly pivotal area of interest in pathology, highlighting the complex relationship between aging processes and cancer development [185].

The regenerative potential of stem cells diminishes with age, affecting tissue repair and regeneration [186]. Stem cell exhaustion is thought to be an important factor in aging and age-related pathologies like musculoskeletal diseases and hematologic diseases.

Aging is associated with chronic low-grade sterile inflammation, often termed “inflammaging [171, 187–190],” which is a significant contributor to many age-related diseases, including cardiovascular diseases, neuroinflammation and neurodegenerative diseases [191–194], sarcopenia [195] and cognitive dysfunction [194, 196, 197].

In pathology, these cellular and molecular mechanisms provide a framework for understanding the complex interplay between aging and disease. By considering these mechanisms, pathologists can gain insights into the onset, progression, and potential therapeutic targets for a wide range of age-related conditions.

## Anti-aging interventions and plasticity of aging

Anti-aging interventions, which specifically target one or more fundamental aging processes, hold immense potential in preventing the development of age-related diseases and altering their trajectories. These therapies, ranging from pharmacological [198–201] to dietary [202], nutraceutical [203], and lifestyle [204] interventions [205–207], aim to slow aging and prevent age-related pathologies. The burgeoning field of pharmacological anti-aging interventions is rapidly evolving, with several promising therapeutic avenues emerging. Among these, senolytics, which selectively target and eliminate senescent cells, are at the forefront [176, 180, 181, 208–210]. These agents offer potential in mitigating the detrimental effects of cellular senescence, a key contributor to age-related diseases and dysfunctions. Another area of significant translational relevance is mitochondria-targeted treatments [165, 211–213]. Treatments that modulate epigenetic regulation [214, 215] and restore cellular NAD levels [216–226] represent another exciting frontier. Additionally, targeting the mTOR pathway offers significant potential [12]. Inhibitors of the mTOR pathway have shown promising results in extending lifespan and healthspan in various model organisms [15, 113], suggesting their potential applicability in human aging.

As we move into the upcoming decade, we anticipate significant advancements in developing these novel interventions. The role of pathology in this progress is pivotal. With standardized assessment of key biomarkers of aging, pathology will play a crucial role in rigorously testing the efficacy of these interventions. This testing, critical both in preclinical studies and clinical trials, can include methodologies such as using tissue biopsies to assess the impact of these interventions on cellular and molecular levels. The integration of interventions that target cellular and molecular mechanisms of aging into pathological studies not only holds the potential for breakthroughs in disease prevention and treatment but also promises to revolutionize our understanding of disease pathogenesis. This revolution involves introducing geroscience concepts into pathology, further bridging the gap between aging research and clinical practice.

## Novel tools and experimental models from geroscience

Geroscience has developed an array of tools and models over the past decade that can be adapted to pathology research. One key area is the use of aging models to study diseases. Traditionally, research on age-related diseases like cancer, stroke, hypertension, and myocardial infarction has been regularly conducted using young animal models. However, these models often fail to capture the complexities of human diseases, which typically manifest in older populations. By employing aging models, pathologists can better understand how these diseases develop and progress in an aging body, leading to more accurate representations and insights.

### The shift to age-relevant disease models

The integration of age-appropriate models in pathological studies is crucial. Diseases in older adults often present differently than in younger individuals, both in terms of symptoms, severity and underlying pathophysiology. By using preclinical models that more closely resemble the chronological age at which these diseases commonly occur in humans, researchers can uncover new aspects of disease mechanisms that were previously obscured in younger models. This shift could lead to the development of more effective diagnostic tools and treatments that are tailored to the older population actually affected by these diseases.

The COVID-19 pandemic has starkly highlighted the importance of age-relevant disease models in pathology [227, 228]. COVID-19’s associated pathologies extend beyond respiratory symptoms, encompassing a range of complications including lung injury, systemic microvascular damage, cardiac abnormalities, and neurological manifestations [229–239]. The consequences of infection with SARS-CoV-2 have been particularly severe in older adults [240, 241] and in individuals with accelerated biological aging due to factors such as obesity [242–254]. Geroscience has been instrumental in uncovering how specific age-related mechanisms may contribute to this increased disease severity [244–246, 248, 249, 252, 255, 256]. For example, aging-related changes in the immune system, known as immunosenescence, and inflammaging, are believed to play key roles in the heightened vulnerability and severity of COVID-19 in the older population [257–262]. Understanding the unique impact of COVID-19 on older adults is essential, making the use of age-relevant models in research crucial.

### Utilizing models targeting aging mechanisms in pathology

Pathology stands to gain substantially from integrating models that target aging mechanisms, a key area of focus in geroscience research. Cellular senescence serves as a prime example of an aging process with profound implications for disease development and progression. Over the past decade, a variety of murine models have been developed, allowing for the precise detection and elimination of senescent cells [183, 263–266]. This advancement has shed light on the role of senescent cells in the pathogenesis of numerous age-related diseases. By adopting these geroscience-derived models, pathologists can broaden their investigative scope, targeting a wider array of diseases. This approach not only deepens our understanding of the cellular and molecular foundations of these conditions but also paves the way for more targeted diagnostic and therapeutic strategies in a range of age-related diseases. Fields such as cancer research [263] and musculoskeletal [265–267], ophthalmological [268, 269], otological, and hematological [6, 264] research in pathology are prime examples where an interdisciplinary approach, integrating a geroscience perspective, could lead to significant breakthroughs. By embracing this cross-disciplinary methodology, pathologists can uncover novel insights and develop groundbreaking treatments in these specialized areas, all deeply influenced by the aging process.

### Epigenetic clocks and biological age assessment

Methods developed to assess mechanisms of aging and biological age [100, 270–275], such as epigenetic clocks [47, 94, 100–102, 276–281], offer valuable tools for pathological studies. These methods can be used to determine the biological age of tissues and cells, which may differ from chronological age. Incorporating these measurements into pathological research can provide new insights into the relationship between aging and disease, and potentially uncover new biomarkers for early detection and intervention.

## Conclusion

In summary, geroscience offers a transformative perspective for pathology, bridging the gap between aging processes and disease pathogenesis. By adopting age-appropriate disease models and preclinical models that target specific aging mechanisms, utilizing tools to assess biological aging, and embracing an interdisciplinary approach, pathology can greatly enhance its understanding and treatment of age-related diseases. This integration marks a pivotal shift in the approach to disease research, offering the promise of effective and targeted interventions for the aging population. As the field of geroscience continues to evolve, it will undoubtedly become an integral part of pathology, offering new insights and diagnostic protocols.

The National Institute on Aging (NIA) in the United States has established a vital network of core facilities known as the Nathan Shock Centers of Excellence in the Biology of Aging [282–290]. These centers offer specialized services on a fee-for-service basis to researchers aiming to characterize specific mechanisms of aging and assess biological age. Such initiatives play a crucial role in fostering the incorporation of geroscience concepts into pathological research. By providing access to advanced technologies and expertise, these centers enable researchers to delve deeper into the complexities of aging at a molecular and cellular level [282–290]. This approach not only enhances our understanding of the aging process but also accelerates the development of targeted interventions for age-related diseases. The success of the Nathan Shock Centers serves as a model that could be replicated by other countries, particularly in the European Union, to promote global collaboration and advancement in aging research. By establishing similar facilities, there can be a concerted effort towards integrating geroscience more broadly into various fields of study, including pathology, thereby paving the way for groundbreaking discoveries and innovations in the science of aging and pathology and pathophysiology of age-related diseases.
